# Overcome the barriers of the skin: exosome therapy

**DOI:** 10.1186/s40824-021-00224-8

**Published:** 2021-07-03

**Authors:** Gi Hoon Yang, Yoon Bum Lee, Donggu Kang, Eunjeong Choi, Yoonju Nam, Kyoung Ho Lee, Hi-Jin You, Hyo Jin Kang, Sang Hyun An, Hojun Jeon

**Affiliations:** 1 Research Institute of Additive Manufacturing and Regenerative Medicine, Baobab Healthcare Inc., 55 Hanyangdaehak-Ro, Ansan-si, Gyeonggi-Do 15588 South Korea; 2 Laboratory Animal Center, Daegu-Gyeongbuk Medical Innovation Foundation (DGMIF), 80 Cheombok-ro, Dong-gu, Daegu, 41061 South Korea; 3 Department of Plastic Surgery, Korea University Ansan Hospital, 123 Jeokgeum-ro, Danwon-gu, Ansan-si, Gyeonggi-Do 15355 South Korea; 4 Biomedical Research Center, Korea University Ansan Hospital, 123 Jeokgeum-ro, Danwon-gu, Ansan-si, Gyeonggi-Do 15355 South Korea

**Keywords:** Exosomes, Biomarker, Skin defects, Skin treatment

## Abstract

Exosomes are nano-sized cargos with a lipid bilayer structure carrying diverse biomolecules including lipids, proteins, and nucleic acids. These small vesicles are secreted by most types of cells to communicate with each other. Since exosomes circulate through bodily fluids, they can transfer information not only to local cells but also to remote cells. Therefore, exosomes are considered potential biomarkers for various treatments. Recently, studies have shown the efficacy of exosomes in skin defects such as aging, atopic dermatitis, and wounds. Also, exosomes are being studied to be used as ingredients in commercialized skin treatment products. In this review, we discussed the need for exosomes in skin therapy together with the current challenges. Moreover, the functional roles of exosomes in terms of skin treatment and regeneration are overviewed. Finally, we highlighted the major limitations and the future perspective in exosome engineering.

## Background

In the early 2000s, mesenchymal stem cell therapy had risen as an alternative for the treatment of various defects and diseases [1]. Mesenchymal stem cells (MSCs) are widely used in clinical trials not only due to their multipotency but also the ease of accessibility, expansion, and isolation from various adult tissues such as skin, placenta, cord blood, cord tissue, adipose tissue, dental pulp, testicles, and brain [2–9]. However, the safety and efficacy of stem cell therapy have been controversial [10]. Furthermore, several studies have shown that MSCs themselves were not engaged in the therapeutic process [11]. The stem cells injected in the defected site showed low cell viability and low numbers of cells tend to fuse with the recipient cells in the host tissue [12–14]. Instead, recent studies have revealed that the therapeutic efficacy of MSCs was beneficial through the release of biological molecules. These biologically active factors are secreted in the form of micro to nanosized particles called extracellular vesicles (EVs) by cells [15]. EVs contain signals in the form of lipids, proteins, and nucleic acids which can be exchanged between cells engaged in the regulation of physiological and pathological activities. Depending on the size of the particle, EVs can be divided into exosomes (40 to 100 nm), microvesicles (150 to 1000 nm), and apoptotic bodies (> 1000 nm) (Table 1) [16–18]. Moreover, the release mechanisms are different in which microvesicles are released directly from the cell membrane, while exosomes are released via the fusion of the multivesicular bodies (MVBs) and the plasma membrane. In this review, we will focus on the smaller particles in the EVs, the exosomes.

**Table 1:** Characteristics of exosomes, microvesicles, and apoptotic bodies

	Exosomes	Microvesicles	Apoptotic bodies	Ref
**Size**	40–100 nm	150–1000 nm	> 1000 nm	[16–18]
**Composition**	ProteinsLipids RNAs	ProteinsLipids RNAs	DNA fragments Degraded proteins Micronuclei Cell organelles	[19, 20]
**Origin**	Multivesicular bodies	Plasma membrane	Plasma membrane, cellular fragments	[21]
**Release**	Exocytosis of MVBs	Budding from plasma membrane	Cell shrinkage and death	[21]

## Exosomes: intracellular communication tool

### History of exosomes

The first observation of exosomes was as early as in 1960s, while not much was known [22]. More findings regarding to exosomes were obtained later in the 1980s. During a study of sheep reticulocyte maturation, the mechanism of exosome formation was revealed [23]. The study showed that small vesicles were formed inside endosomes and then released into the extracellular environment during exocytosis. Years later, a study proposed that exosomes were small shuttles containing mRNAs and microRNAs (miRNAs) which enable remote genetic communication [24]. Since then, exosome has unveiled a new paradigm in various therapeutic fields.

### Exosome biogenesis

**Fig. 1:**
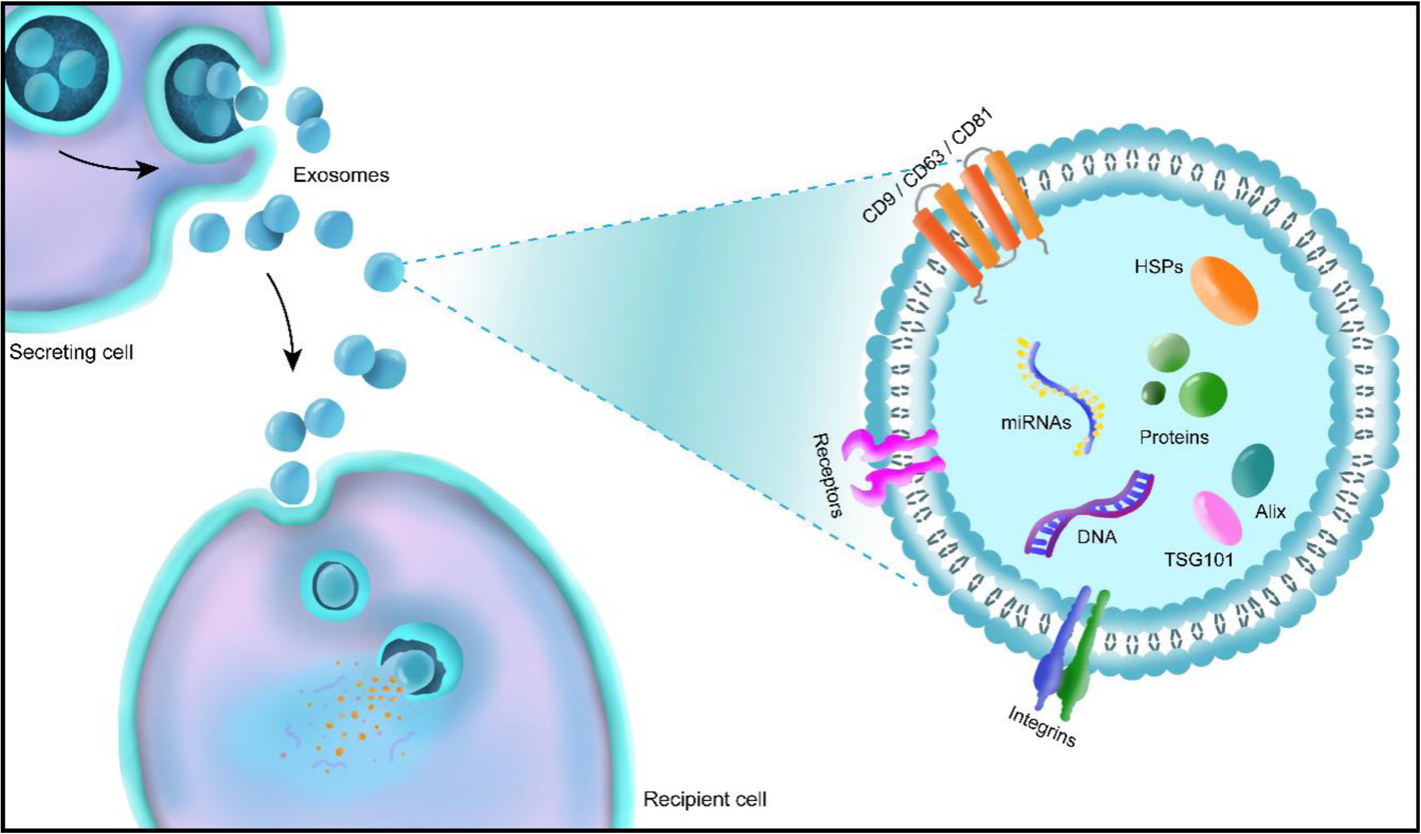
Schematical illustration of the exosome biogenesis

In general, exosomes are characterized by exosomal markers including CD9, CD63, CD81, HSP60, HSP70, HSP90, Alix, and TSG 101 [25]. CD9, CD63, and CD81 are part of the tetraspanin family associated with regulation of various cellular responses such as cell-to-cell communication, cell fusion, tumor cell metastasis, cell motility, and signal transduction (Fig. 1) [26–36]. Since exosomes contain signals in form of proteins, mRNA, and miRNAs, distinct signaling cues can be obtained depending on the cell types. Recently, MSCs-derived exosomes have gained attention due to their vast capacities for therapeutic efficacy. Although the RNAs carried by the exosomes are dependent on the tissue source from which MSCs are extracted, no correlation was found between the therapeutic efficacy of exosomes and the tissue source [37, 38]. Therefore, MSCs derived exosomes were intensively studied for the treatment of various defects and illnesses.

### Exosome isolation techniques

Since it is crucial to isolate high quality exosomes, various isolation techniques have been introduced. After the isolation process, the exosomes must preserve their biological functions. Some of the isolation methods are ultracentrifugation (UC)-, ultrafiltration (UF)-, precipitation (PT)-, chromatography (CT),- and microfluidic (MF)-based techniques [39]. In ultracentrifugation-based methods, the exosomes are isolated from body fluids or cell culture media via a centrifugal force which separates the constituents according to their density, size, and shape. Centrifugation is widely used to separate and purify solutions. UC-based techniques are relatively simple and preserves the physiochemical properties of the exosomes after isolation. However, this technique requires specialized equipment and low purity is another downside [40]. In case of UF-based methods, the exosomes are isolated through ultrafine membranes with different molecular weight cut-off. Compared to the UC-based methods, the processing time is shorter and no special equipment is required. However, clogging and trapping of the particles leading to a low purity is a noticeable limitation [40]. Next is the PT-based methods which is based on the lowering the solubility of the exosomes by adding highly hydrophilic polymers to the solution. This process induces precipitation for an easy collection of the exosomes. PT-based methods provide high reproducibility with high yield. Moreover, this method only requires lab-bench equipment. However, PT-based methods can cause exosome aggregation [40]. Another technique is the CT-based method which separates particles with different sizes by adding porous materials to the exosome containing solution [41]. This method has the advantage of preserving the natural biological state of the exosomes. Other benefits are good reproducibility, high purity, and short preparation time. However, contaminants (protein aggregates and lipoproteins) with the size range within that of the exosomes are also isolated. Finally, the miniaturized microfluidic devices used in MF-based methods provide a rapid isolation process and a real-time analysis of the exosomes. However, these methods have shown low reproducibility and require multi-step integrated platforms [40]. Most of the currently available methods lack the ability to efficiently isolate high-quality exosomes in large scale [42]. Accordingly, some exosome companies use combinational isolation processes to improve the throughput of high-quality exosomes such as combination of UC and UF methods.

### Exosomes for therapeutic applications

For the use of exosomes in therapeutic applications, high extraction efficiency and mass production of high-quality exosomes are crucial. To achieve this, upstream (cell culture) and downstream (isolation) manufacturing processes are of great importance. The mass production of exosomes strongly relies on the cell culture technique. Current scale-up manufacturing systems are based on 2D cell culture methods including multilayer flask processes. Various studies have shown that 3D-based cell culture resulted in increased particle number and enhanced quality compared to 2D-based culture. Some examples of 3D-based cell culturing methods include the use of 3D scaffolds, bioreactors, and spheroids [43–45].

To obtain high-quality exosomes, the isolation techniques should provide high yield with high degree of purity. Antounians et al. compared and analyzed different isolation techniques including UC, PT, and CT-based methods [46]. Here, PT (ExoQuick, Total Exosome Isolation Reagent, and Exo-PREP) and CT (qEV) methods are commercially available. Accordingly, the smallest EVs were obtained when isolated using qEV. However, qEV resulted in the lowest EV number, while UC, Exo-PREP, and ExoQuick provided the greatest number of EVs. In terms of protein content, Exo-PREP extracts showed the greatest content. On the other hand, qEV isolations resulted in the lowest protein content. The research group also revealed that the cell survival was dose dependent using the isolated EVs in a lung epithelial injury model. Moreover, other studies also showed that the regenerative effect of exosomes was found to be dose dependent [47–50].

### Applicability of exosomes

Owing to their various benefits, exosomes have been widely utilized in various therapeutic applications. Exosomes have the ability to carry biomolecules including proteins, DNAs, and RNAs intrinsically. Therefore, depending on the cell type, we can obtain exosomes with distinct therapeutic abilities. For example, exosome secreted by cardiac progenitor cells prevented myocardial cells against oxidative stress-related apoptosis [51]. Another study demonstrated that dermal papilla cells-derived exosomes promoted the development of hair follicles [52]. Furthermore, exosomes can act as a cargo for drug delivery systems (DDS). Exosomes have extremely low immunogenicity with high hemocompatibility. The precise targeting of exosomes is another advantage favoring the use in DDS. In addition, they are highly stable in body fluids and can fuse with recipient cells easily. Finally, exogenous drugs can be loaded into exosomes to target a specific disease. Kim et al. successfully loaded cancer-related miRNA, let7c-5p, into human embryonic kidney 293 T (HEK293T) cells-derived exosomes for breast cancer therapy [53]. As a result, let7c-5p efficiently suppressed cancer cell proliferation and migration. For these reasons, exosomes have shown their potential in treating various diseases. In particular, this review will discuss the need for exosome therapy in skin treatments.

## Skin therapy and regeneration

### Skin tissue

**Fig. 2:**
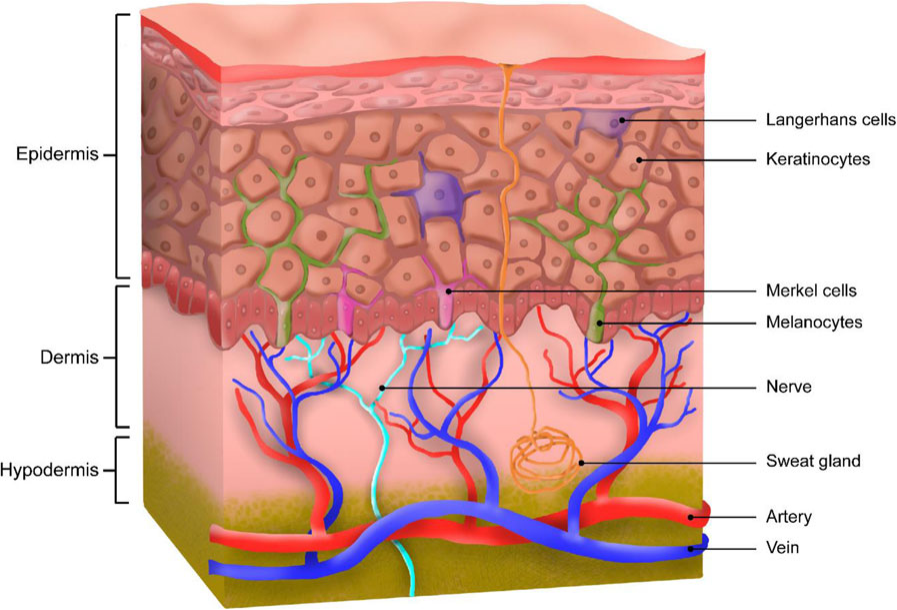
Human skin anatomy showing the three layers: epidermis, dermis, and hypodermis

Skin is the largest organ in the human body acting as a protective barrier between the external and internal environment [54]. This sensory organ is also responsible for the regulation of body temperature and moist release into the environment. The skin thickness varies depending on the body area such as underneath the eyes, palms of the hands, upper back, etc. In an anatomical perspective, the human skin has an intricate structure composed of three layers: epidermis, dermis, and hypodermis each buildup of different cell types (Fig. 2). The types of cells and their functions are listed in Table 2.

**Table 2:** List of skin cells and their functions

	Cells	Function	Ref
**Epidermis**	Keratinocytes Melanocytes Langerhans cells Merkel cells	Preservation of immune barrier Skin pigmentation Adaptive immune responses Mechanoreceptors	[55] [56] [57] [58]
**Dermis**	Fibroblasts Mast cells	Synthesizing and depositing ECM component Immune and inflammatory responses	[59] [59]
**Hypodermis**	Adipocytes	Energy storage, endocrine, nervous, and immune function	[60]

### The need for skin therapy

**Fig. 3:**
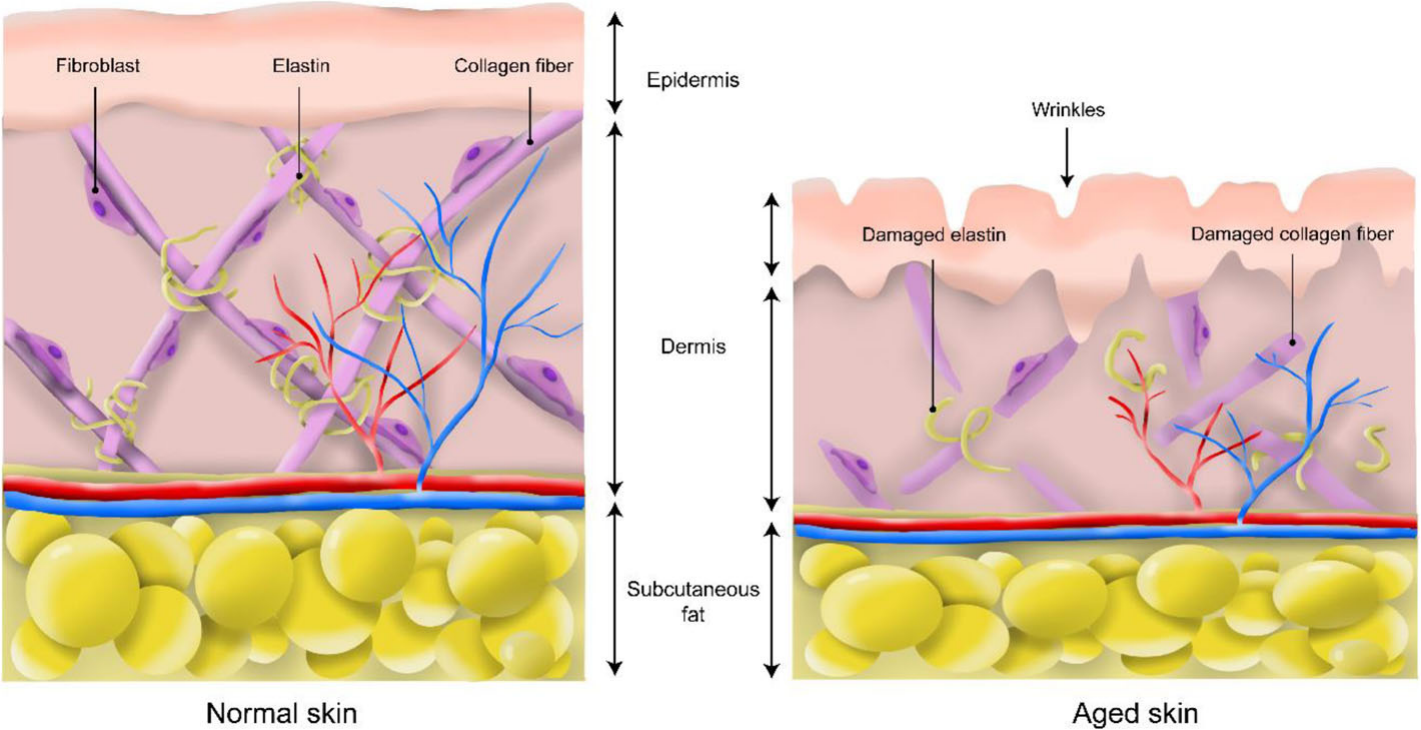
The structures of the skin before and after aging

As life progresses, physical changes in the skin are the first observation evidencing organismal aging. Since the ancient times, people have strived to improve the quality of their skin through anti-aging treatments. In the present days, the skin is an important part in social lives boosting self-esteem and self-consistency. Therefore, various anti-aging treatments have been introduced in the cosmetic industry [61]. The skin aging is determined by extrinsic (environmental aspects: UV exposure, air pollution, smoking, and nutritional deficiency) and intrinsic (cellular and hormonal changes) factors progressing the loss of functionality and regenerative potential [62]. Especially, intrinsic skin aging is a time-dependent and inevitable process. One of the factors damaging the skin is the formation of reactive oxygen species (ROS) which hastens the aging process of the skin [63]. As a result, the epidermal layer becomes thinner attributable to keratinocyte atrophy. This leads to water loss increasing the dryness and coarseness of the skin [64]. Another factor is acceleration of telomere attrition, shortening of the telomers, causing the skin to age. Telomeres are protein-DNA structures protecting the ends of chromosomes avoiding the loss of terminal DNA sequences. As the telomeres shortens over time, cells become senescent. Cellular senescence induces changes in the different layers of the skin and aggravate skin aging. However, preventing telomeres from shortening might have severe consequences such as cancer formation [65]. Furthermore, the extracellular matrix (ECM) components including collagen and elastin reduces due to increased matrix metalloproteinase (MMP) expression causing loss of structural integrity (decreased tensile strength and elasticity) [66]. Correspondingly, the junction between the dermis and epidermis decreases leading to thinning of the skin. Therefore, formation of wrinkles is observed in aging skin (Fig. 3) [67]. All of these factors gradually alter the intracellular environment of the skin causing changes in the skin morphology.

**Fig. 4:**
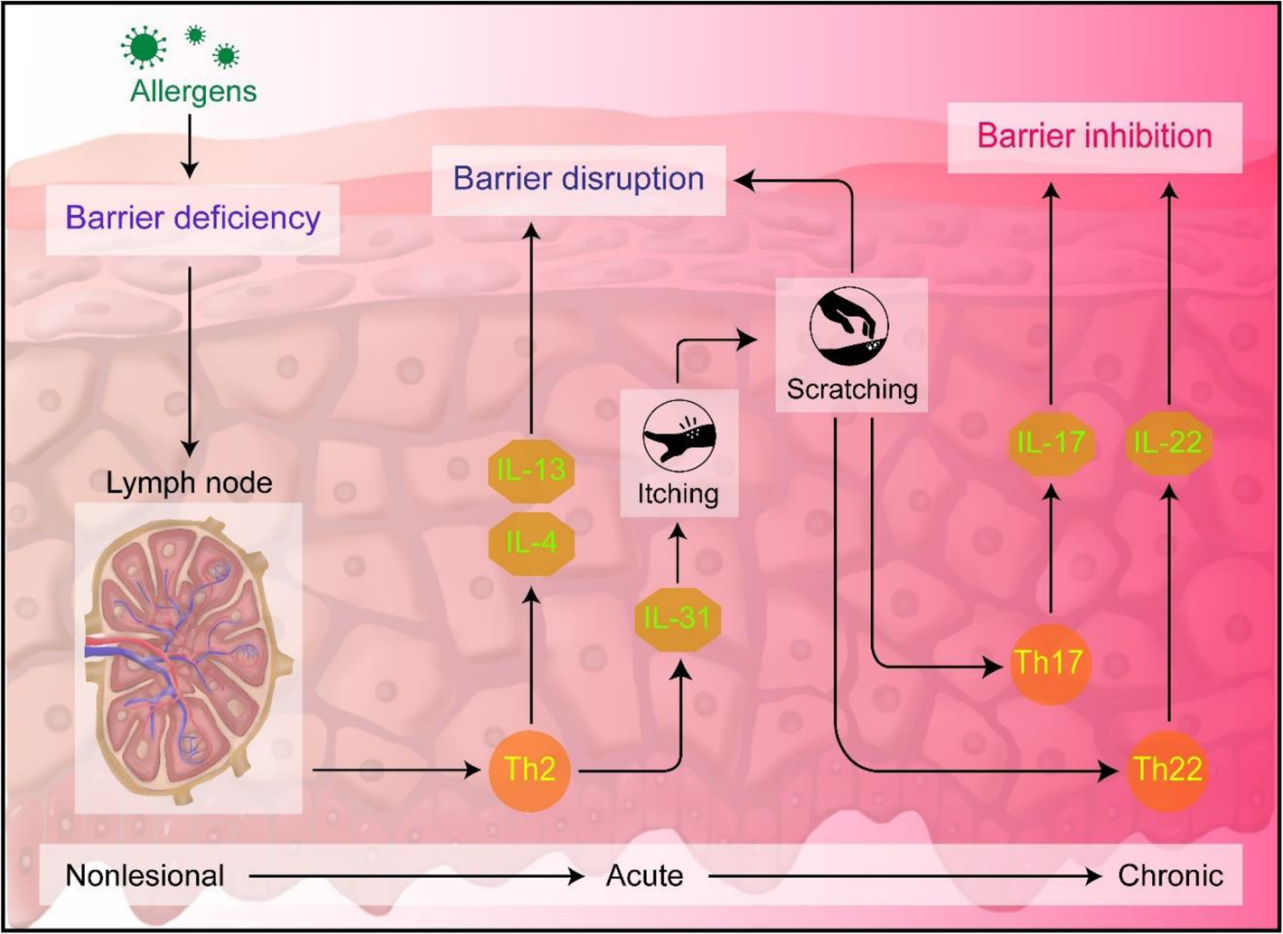
Illustration of immune pathways and the corresponding responses in atopic dermatitis process

Atopic dermatitis (AD) is one of the most common inflammatory skin disorder in children in which the major symptom of AD is pruritus which can cause anxiety, depression or social avoidance impairing the quality of life [68]. Moreover, AD increases the risk of developing other diseases including allergic rhinitis or asthma [69]. The main causes of AD are thought to be the dysfunction of the skin barrier and immune responses [70]. Figure 4 illustrates the key aspects in the pathogenesis of AD. In brief, the predominance of T helper (Th) lymphocytes 2 responses in AD initiating the disruption of barrier function and immune dysfunction through the expression of cytokines including interleukin (IL)-4 and IL-13 [71]. In specific, the key inflammatory cytokines IL-4 and IL-13 are responsible for the downregulation of filaggrin (FLG) expression which is involved in the formation of epidermal barrier [72]. This causes increase in allergic reactions. Another cytokine expressed by Th2 is IL-31 correlated to the development of pruritus [73]. This cytokine can bind to the cognate receptors on neurons and activate itch sensory neurons through Janus kinase (JAK)-signal transducer and activator of transcription (STAT) pathway [74]. Scratching intensifies the allergic reactions leading to expression of IL-17 and IL-22 which are considered as the main drivers of epidermal hyperproliferation by Th17 and Th22, respectively [75]. Eventually, the process exacerbates and develop into a chronic state, psoriasis. Until now, no effective treatments for AD have been developed.

**Fig. 5:**
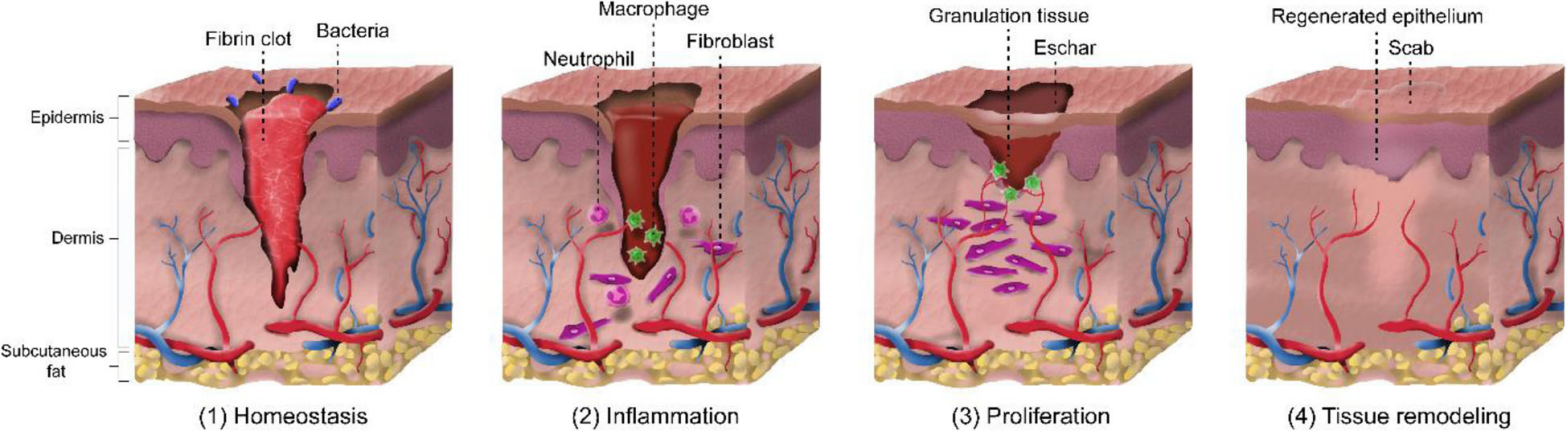
Wound healing process including the four stages

The skin has self-healing ability capable of healing wounds through a cascade of events: (1) homeostasis, (2) inflammation, (3) proliferation, and (4) tissue remodeling [76]. This is a continuum process until it is interrupted, aberrated, or prolonged leading to a delayed wound healing or formation of chronic wound. Figure 5 shows the graphical illustration of the normal wound healing process. The process all begins with homeostasis in the first phase characterized with vascular constriction and fibrin clot formation at the wound site [77, 78]. In this step, the purpose is to prevent exsanguination protecting the vascular system. After, wound healing related molecules (transforming growth factor (TGF)-β, platelet-derived growth factor (PDGF), fibroblast growth factor (FGF), and epidermal growth factor (EGF)) are released from the clot and surrounding tissue which attracts neutrophils, macrophages, endothelial cells, and fibroblasts initiating the inflammatory response [76, 77, 79, 80]. In the next phase, cells including fibroblasts and endothelial cells engage in the synthesis of collagen, capillary growth support, and formation of granulation tissue at the wound site. Finally, the wound healing process undergoes the remodeling phase in which the granulation tissue grows into an avascular scar. However, delayed or interrupt wound healing process results in scar formation in the skin. Although the mechanism underlying the repair of the skin is well-known, no clear treatments are available yet.

### Current skin therapies

Generally, the treatment for the above-mentioned skin deficiencies (aging, AD, and wound healing) is based on topical or invasive strategies using chemical, biological, and physical agents. Table 3 shows the various strategies for skin treatment purposes.

**Table 3:** Current skin therapy strategies

Anti-aging	Non-invasive	Cosmetics (moisturizers, creams, sunscreens, antioxidant serums) Chemical peels Phototherapies
	Invasive	Micro-needling Injectables (Botox, skin boosters, stem cell therapies) Thread lifts
**Atopic dermatitis**	No prescription	Moisturizers (lotions, creams) Oral antihistamines
	Prescription	Topical steroids Topical calcineurin inhibitors Injectable anti-inflammatories Oral medications
**Wound healing**	Non-chronic	Dressing (Hydrocolloids, hydrogels, alginate, collagen) Ointments Sprays
	Chronic	Biological skin substitutes Biosynthetic skin substitutes Synthetic skin substitutes

Current anti-aging strategies can be categorized into non-invasive and invasive procedures. The non-invasive skin treatment comes in the form of cosmetics, chemical peels, and phototherapy. To enhance the protection from oxidative stresses initiated by ROS, antioxidants and cell regulatory agents including growth factors and peptides are used as constituents [81–87]. As an example, retinol known as vitamin A has been extensively used in cosmetics in forms of topical serums, creams, oils, etc. due to their beneficial effects towards skin treatments such as anti-aging effects [88–91]. Retinol which is fat-soluble penetrates the skin through stratum corneum and reaches the skin dermis. Once it penetrates the skin, retinol is responsible for strengthening the epidermal layer and inhibit the activity of metalloproteinases which are involved in collagen degradation [92, 93]. One of the widely used physical therapy is laser treatment capable of production of collagen and elastin, dermal remodeling, and reduction of pigment and erythema [94]. In invasive therapy, micro-needling have gained attention in skin therapy which allows drug molecules to penetrate the skin [95]. The microneedles create micro-sized pathways for a direct drug delivery into the skin skipping the skin barrier. Some of the cosmetic ingredients used in micro-needling therapy are peptides, growth factors, and acids [96–98]. Other invasive procedures include injectables and thread lift. Compared to the non-invasive products, invasive procedures provide more effective and rapid anti-aging outcomes which usually lasts for a short amount of period.

Until now, there is no complete cure for AD available. The current over-the-counter products add moisture to the skin or reduce the itching symptom. On the other hand, majority of the prescription products include steroids or calcineurin inhibitors to provide anti-inflammatory effects. Likewise, the complete healing of AD is not possible using these products.

For an ideal wound healing, the product should provide proper oxygen permeability, protect from contaminants and moisture-loss, and mimic the skin structure [99]. Especially, for chronic wound management, skin substitutes are required. However, current available skin substitutes possess some limitations such as scar development, costly process, immune rejection, insufficient structure, etc. [100].

Although current skin therapy has shown some cosmeceutical effects, most of the available skin treatment products lack the capacity to heal the skin completely.

## Future ingredient for skin treatment: exosomes

**Fig. 6:**
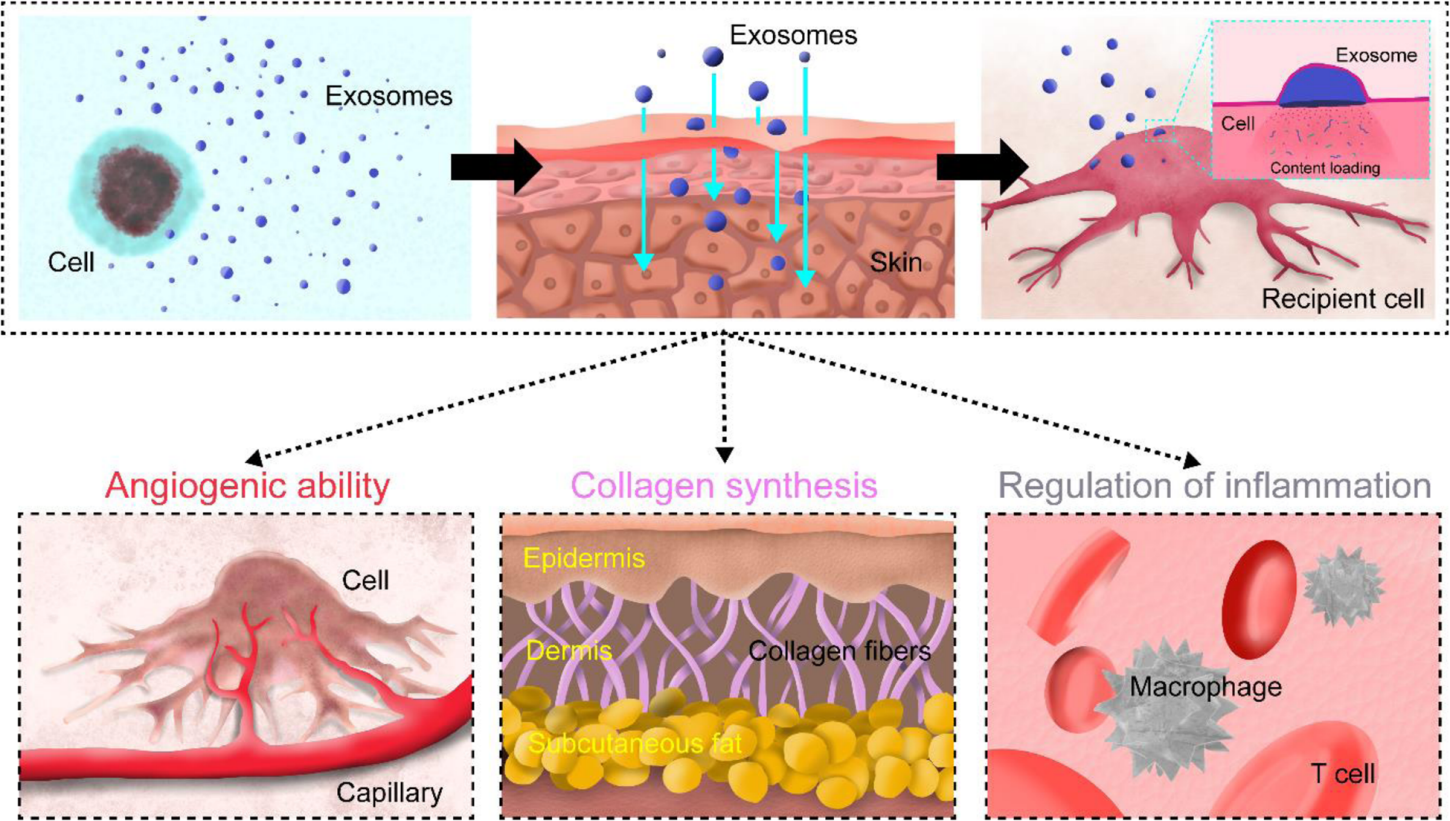
Schematic representation showing skin regenerative abilities of exosomes

In recent years, the effects of exosomes on various skin defects have been extensively studied. The key benefits of exosomes are the high stability, non-immune rejection, and direct stimulation of target cells [101, 102]. Especially, the miRNAs contained in the exosomes are engaged in the regulation of various cellular responses by binding to the 3′-untranslated region [103]. Therefore, a single ingredient (exosomes) can contribute to multiple therapeutic effects (Fig. 6).

### The regenerative potency of exosomes

#### Angiogenic ability

As the skin ages, various changes occur such as decrease in the collagen type-I content, disorganization of the capillary network, denaturation of elastic fibers etc. [63]. These degenerative changes may result in decreased vessel structures in the skin tissue. A proper vascularization is an essential part for a healthy skin which provides nutrient supply to the skin cells to maintain their activities. Therefore, one solution to aging skin may be solved by increasing the vascular number. Moreover, vascularization plays an important role in wound healing [104]. Various studies have shown the angiogenic properties of exosomes. In a study, exosomes secreted by human adipose-derived MSCs were examined on human umbilical vein endothelial cells (HUVECs) for their effect on angiogenesis [105]. This study demonstrated the potential role of a microRNA (miR-125a) in the exosome in angiogenesis. Overexpression of miR-125a enhanced angiogenesis in HUVECs by targeting DLL4, a ligand of Notch signaling pathway. In another study, dermal endothelial cells isolated from young and old volunteers were compared [106]. As a result, miR-100, miR-126, and miR-223 detected in both groups were significantly downregulated in the group with old volunteers. Among these microRNAs, miR-126 is known to be engaged in the protection of blood vessels and neovascularization [107]. Furthermore, miR-214 found in the exosomes extracted from the human microvascular endothelial cell line (HMEC-1) showed angiogenic potential [108]. Senescent cells can absorb miR-214 from exosomes secreted by neighboring cells to exit the senescent state. Also, the exosomal miR-214 may modulate the formation of blood vessels.

#### Collagen synthesis

As mentioned before, the dermal layer is damaged due to fragmentation of collagen type-I and elastic fibers in aged skin. Therefore, rebuilding the dermal structure might result in anti-aging and wound healing of the skin. MiR-21 is known to promote keratinocyte migration and re-epithelialization in skin wound healing [109]. Another studied revealed that miR-21 enhanced the wound contraction and collagen deposition [110]. As an example, exosomes derived from human dermal fibroblasts spheroids were evaluated for anti-aging purposes [111]. For this study, the exosomes were tested on UVB-irradiated nude mice. The exosome treated groups showed enhanced collagen synthesis and decreased MMP-1 production. Specifically, TIMP-1 and TGF-β which regulate MMP suppression were upregulated, while miR-196a was downregulated resulting in increased collagen synthesis. Moreover, deep and wide wrinkles due to the UVB irradiation became more superficial and thinner after the exosome treatment. Kim et al. examined the effects of exosomes derived from human umbilical cord blood-derived mesenchymal stem cells (UCB-MSCs) for skin rejuvenation [112]. Among various growth factors detected in the exosomes, EGF was found in greater concentrations. When human dermal fibroblasts (HDFs) were exposed to the exosomes, collagen and elastin production was enhanced. Thus, UCB-MSCs-derived exosomes can be used to rebuild the dermal layer.

#### Regulation of inflammation

AD is an inflammatory skin disease caused by skin barrier defect [113]. Exosomes might be used as a therapeutic ingredient to treat AD. Cho et al. investigated the effect of adipose tissue-derived mesenchymal stem cell (ASC)-derived exosomes on AD treatment [114]. ASC-exosomes downregulated the expression of inflammatory cytokines such as IL-4, IL-23, IL-31, and TNF-α in a mouse AD model. Therefore, a decreased AD symptom was observed in the mouse model in a dose-dependent manner. Another study suggested that MSC derived exosomes has the ability to balance Th1 and Th2 and inhibit local inflammatory reaction which can alleviate AD symptoms [115]. Since the immune balance is the key factor in AD, Wu et al. studied the role of miR-210 which is highly expressed in patients with psoriasis [116]. They reported in their study that miR-210 induced Th17 and Th1 cell differentiation, while repressing STAT6 and LYN gene expression resulting in inhibition Th2 differentiation. This revealed to contribute to immune balance in psoriasis.

### Various studies on skin regeneration using exosomes

Various studies have been reported studying the efficacy of exosomes for skin tissue regeneration. Shafei et al. loaded exosomes extracted from ASCs in an alginate based hydrogel (Alg-EXO) for skin therapeutic purposes [117]. A rat wound model was used to observe the feasibility of the exosomes. The alg-EXO group was compared with non-treated control and alginate only group. The in vivo results showed that the alg-EXO group significantly improved the wound closure compared to the other groups after 15 days. The exosomes seem to induce collagen deposition and vascularization enhancing the wound healing process. In another study, an injectable self-healing hydrogel (FHE) composed of Pluronic F127, oxidative hyaluronic acid (OHA), and poly-ε-L-lysine (EPL) was developed with ASCs-derived exosomes releasing ability [118]. The released exosomes reduced the healing time of the wound in a diabetic mouse wound model. This was possible due to the enhanced cell proliferation, faster granulation tissue formation, re-epithelialization, and collagen remodeling within the wound sites. Therefore, less scar tissue was observed with improved wound healing. Moreover, Shi et al. prepared a chitosan/silk scaffold loaded with gingival MSCs-derived exosomes for skin regeneration [119]. Although the scaffold alone showed great wound healing effects in the diabetic rat skin model, exosome containing scaffold showed significantly greater healing ability. The results showed that the exosomes enhanced re-epithelialization and ECM deposition/remodeling in the defect site. In addition, Zhao et al. developed a gelatin methacryloyl (GelMA) hydrogel based wound dressing consisting of HUVECs-derived exosomes and applied it to a wound defect rat model [120]. The results revealed that the exosomes meaningfully accelerated the wound healing via enhanced re-epithelialization, collagen deposition, and angiogenesis.

## Future perspectives and conclusion

### Future perspectives

Exosomes have shown their potential in various tissue regeneration fields including skin. However, there are still some limitations to be solved. First, the precise content and functionality of the miRNA, mRNA, proteins, and lipids in the exosomes are still unanswered [121]. It is extremely important to clarify the intrinsically packed natural ingredients in the nano-sized cargo. We have to be able to avoid the use of exosomes with unwanted or harmful substances including damage-associated molecular patterns (DAMPs). DAMPs are known as alarmins which are released from injured tissues initiating the immune system [122]. Triggering inflammatory responses in an inadequate situation may cause diseases such as autoimmune diseases, cardiovascular diseases, and neurodegenerative diseases [123]. Therefore, standardization, scalability, and validation are still the roadblocks for clinical uses. Second, despite the advances in exosome isolation techniques, no gold standard is yet established [124]. The isolation procedure cost and complexity should be reduced, while the exosome purity should be increased. Third, for the use in therapeutic applications, mass-production of high-quality exosomes should be possible. However, this is challenging with the current culture and isolation methods. To improve the efficiency, a multi-functional system should be developed with highly efficient isolation technique and real-time quantification and analysis technology. Fourth, personalized skin treatment products might be developed using exosomes in the future. Depending on the skin type, condition, and defect, the amount and type of the exosome can be selected. To do this, preservation methods of exosomes should be considered. To maintain the proteins and RNAs within, exosomes should be stored below − 70 °C [125]. Other storage method is the freeze-drying technique in which using cryoprotectants is recommended [126]. However, long-term preservation using these methods is still not clarified to be used in diagnosis and therapeutic applications. Finally, exosomes might be the key element in the field of medicine one day. Various studies are ongoing to find the cure for not only cancer but other incurable diseases such as Parkinson’s disease, Alzheimer’s disease, and amyotrophic lateral sclerosis (ALS) [127–132]. Another potential approach using exosomes is vaccine development [133].

## Conclusion

Exosomes emerged as the beneficial alternative for MSCs in the field of regenerative medicine. These potential nano-carriers exhibit various therapeutic characteristics for skin treatments. The curiosity towards exosomes has acted as the driving force behind the progress and development. Since then, thousands of studies have been published and exosomes are being researched for purposes of commercialization. This review discussed about exosomes as the potential candidate for skin treatments. Exosomes have several abilities beneficial for skin tissue regeneration: (1) angiogenic ability, (2) collagen synthesis, and (3) regulation of inflammation. Using exosomes as the ingredient, current limitations of the skin treatments can be overcome. Moreover, these fascinating biomaterials can be integrated into various fields such as cancer treatment and vaccines. Hopefully, exosomes will provide a new platform to cure various diseases and cancers in the near future.

## Data Availability

Not applicable.

## Abbreviations

MSCs: Mesenchymal stem cells
EVs: Extracellular vesicles
MVBs: Multivesicular bodies
miRNAs: microRNAs
UC: Ultracentrifugation
UF: Ultrafiltration
PT: Precipitation
MF: Microfluidic
DDS: Drug delivery systems
HEK293T: Human embryonic kidney 293 T
ROS: Reactive oxygen species
ECM: Extracellular matrix
MMP: Matrix metalloproteinase
AD: Atopic dermatitis
Th: T helper
IL: Interleukin
FLG: Filaggrin
JAK: Janus kinase
STAT: Signal transducer and activator of transcription
TGF: Transforming growth factor
PDGF: Platelet-derived growth factor
FGF: Fibroblast growth factor
EGF: Epidermal growth factor
HUVECs: Human umbilical vein endothelial cells
UCB-MSCs: Umbilical cord blood-derived mesenchymal stem cells
HDFs: Human dermal fibroblasts
ASC: Adipose tissue-derived mesenchymal stem cell
Alg-EXO: Exosomes extracted from ASCs in an alginate based hydrogel
FHE: Injectable self-healing hydrogel
OHA: Oxidative hyaluronic acid
EPL: Poly-ε-L-lysine; GelMA: Gelatin methacryloyl
